# Characterisation of a novel A1-specific monoclonal antibody

**DOI:** 10.1038/cddis.2014.519

**Published:** 2014-12-04

**Authors:** M J Lang, M S Brennan, L A O'Reilly, E Ottina, P E Czabotar, E Whitlock, W D Fairlie, L Tai, A Strasser, M J Herold

**Affiliations:** 1 Molecular Genetics of Cancer Division, The Walter and Eliza Hall Institute of Medical Research, Parkville, Victoria, Australia; 2 Department of Medical Biology, The University of Melbourne, Melbourne, Victoria, Australia; 3 Division of Developmental Immunology, Biocenter, Innsbruck Medical University, Innsbruck, Austria

*Dear Editor*,

A1/BFL-1 is the least studied pro-survival BCL-2 family member. This can be largely attributed to the lack of proper tools to study A1/BFL-1 function. Owing to the genomic organisation of the *A1* locus in mice (three expressed *A1* genes and one pseudo-gene, interspersed by unrelated genes)^1^ a knockout is challenging. We generated shRNA transgenic mice in which all functional A1 isoforms were knocked down. In accordance with *A1* mRNA expression studies, we found that A1 is critical for the development and survival of lymphocytes and granulocytes.^2^ As the A1/BFL-1 protein is regulated by ubiquitin-dependent proteasomal degradation, the *A1* mRNA expression data may not truly reflect the A1/BFL-1 protein levels. Previous attempts to generate A1-specific antibodies have failed and commercially available antibodies do not reliably detect the endogenous protein.

To generate A1-specific monoclonal antibodies, we immunised rats with a truncated/mutated A1 protein (delta-C20, P104K)^3^ together with two KLH-conjugated peptides corresponding to central and C-terminal residues of the A1 protein (aa71–84; aa129–154). Screening by ELISA and western blotting identified one monoclonal antibody that detected overexpressed A1-a, A1-b and A1-d, and to a lesser extent overexpressed human homologue BFL-1 (data not shown and Figure 1a). To test whether this antibody could reliably detect endogenous A1, we used the mouse WEHI-231 B lymphoma cells, known to express high levels of this protein.^4^ Western blotting revealed a single band of the molecular weight expected for A1 in untreated WEHI-231 cells (Figure 1b, first lane). Overexpressed A1 protein is highly unstable due to ubiquitin-dependent proteasomal degradation.^5^ To further verify the specificity of the A1 antibody, we tested the impact of protein synthesis inhibition or proteasome inhibition on the protein detected in WEHI-231 cells. As expected, the protein synthesis inhibitor cyclohexamide (CHX) decreased the intensity of the protein band, whereas the proteasome inhibitor (MG132) increased it substantially (Figure 1b). Furthermore, we were able to show that this antibody can be used to immunoprecipitate endogenous A1 protein from lysates of WEHI-231 cells (Figure 1c). Next we examined whether this antibody could also detect endogenous A1 in primary mouse cells. In accordance with previous reports on *A1* mRNA expression,^1^ we could reliably detect A1 protein in haematopoietic tissues, such as the lymph nodes and spleen but not in the heart, kidney, liver or lungs (Figure 1d). Immunohistochemical staining using this antibody showed strong A1 protein staining within cell foci in the germinal centres of lymph nodes of non-immunised mice (Figure 1e). No staining with this antibody against A1 was observed in non-haematopoietic tissues, such as the pancreas or the heart (data not shown). To further validate the specificity of this A1 antibody in primary cells, mouse spleen cells were treated with crosslinking IgM antibodies, a stimulus known to upregulate *A1* mRNA levels in B lymphocytes.^6^ Such BCR (B-cell receptor) stimulation increased the protein band detected by our A1 antibody and its density was further augmented when cells were additionally treated with the proteasome inhibitor MG132 during the last hour of the stimulation (Figure 1f). *A1* mRNA levels are upregulated when bone marrow cells are treated with GM-CSF or when mast cells are stimulated with the calcium ionophore ionomycin.^7, 8^ These stimuli caused strong upregulation of the protein band detected by the A1 antibody and the density of this protein band was further increased by the addition of MG132 during the last hour of stimulation (Figures 1g and h). Finally, we validated the specificity of the antibody by using our A1 knockdown mice. In cells from these animals high GFP levels indicate high levels of *A1* shRNA expression and thus low levels of endogenous A1 protein.^2^ We therefore FACS-sorted GFP-positive and GFP-negative spleen cells and treated them with concanavalin A (ConA), a stimulus known to upregulate *A1* mRNA levels in T cells.^9^ As expected, our antibody detected a protein band of the molecular weight predicted for A1 in ConA-stimulated GFP-negative cells but not in the GFP-positive (i.e. *A1* shRNA expressing) splenocytes (Figure 1i). This confirms the specificity of our A1 antibody.

In conclusion, we present here for the first time a mouse A1-specific monoclonal antibody capable of detecting endogenous A1 protein in cell lines as well as in primary mouse cells. Unfortunately, this antibody does not recognise endogenous levels of human BFL-1 (data not shown). This antibody will be made available commercially.

## Figures and Tables

**Figure 1:**
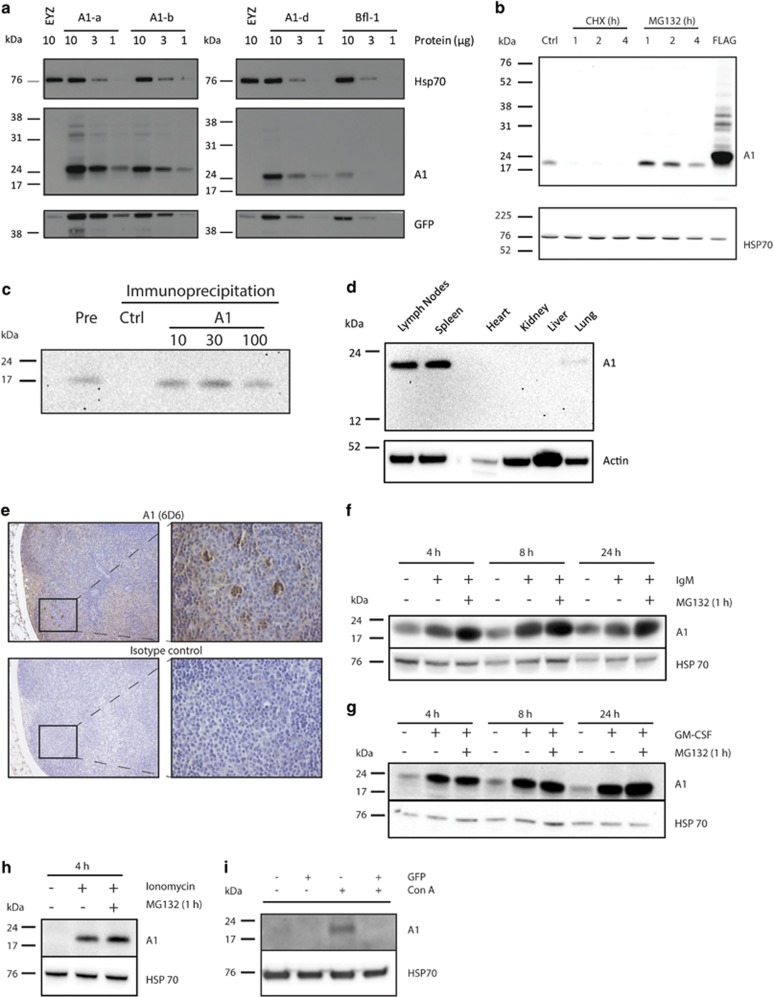
The newly developed A1 antibody reliably detects the endogenous levels of the pro-survival BCL-2 family member A1. (**a**) EYZ (control), A1-a, -b, -d and BFL-1 expression vectors were transiently transfected into 293T cells and protein lysates (total protein amounts as indicated) subjected to western blotting. Probing for HSP70 served as a protein loading control, whereas the GFP detection served as a control for transfection efficiency (GFP is expressed concomitantly with the A1 or BFL-1 proteins from the expression vector). (**b**) Mouse WEHI-231 B lymphoma cells were treated with the protein synthesis inhibitor cyclohexamide (10 *μ*g/ml) or the proteasome inhibitor MG132 (10 *μ*M) for different periods of time (1, 2 or 4 h). WEHI-231 cells transduced with an expression vector encoding FLAG-tagged A1 served as a positive control. (**c**) For immunoprecipitation assays, 5 × 10^6^ mouse WEHI-231 B lymphoma cells were lysed in 500 *μ*l Onyx buffer (20 mM Tris pH 7.4, 135 mM NaCl, 1.5 mM MgCl_2_, 1 mM EGTA, 1% Triton X100, 10% glycerol). Lysates were pre-cleared with 50 *μ*l protein G-sepharose beads and then incubated for 3 h on ice with the indicated dilutions of the A1 antibody (hybridoma culture supernatant) or supernatant from a hybridoma that produces an irrelevant antibody (as a negative control; ctrl); 50 *μ*l protein G-sepharose beads were added during the last hour of incubation. The protein G-sepharose beads were washed four times in Onyx buffer, then incubated with 4xLaemmli gel running buffer and subjected to western blotting. Pre refers to sample taken before immunoprecipitation. (**d**) Protein lysates from the indicated organs were subjected to western blotting. (**e**) 80% Histochoice/20% ethanol-fixed sections from lymph nodes of non-immunised wt (C57BL/6) mice were stained with an antibody to A1 (6D6) or an Ig isotype-matched control antibody (rat IgG2a/*κ*), both used at 50 *μ*g/ml, using conventional immunohistochemistry with DAB as the chromagen and counterstaining with haematoxylin. Photo magnification × 10 and × 40. (**f**) Unsorted spleen cells from wt (C57BL/6) mice were treated for the times indicated with 2 *μ*g/ml anti-IgM F(ab')_2_ fragments. (**g**) Unsorted bone marrow cells from wt (C57BL/6) mice were stimulated for the times indicated with 100 ng/ml GM-CSF. Cells in both (**f**) and (**g**) were also provided with MG132 (10 *μ*M) during the last 1 h of incubation. (**h**) Mast cells were generated by culturing bone marrow cells from wt (C57BL/6) mice for 4 weeks with IL-3 (10 ng/ml) and SCF (12.5 ng/ml); these mast cells were then stimulated for 4 h with 10 *μ*g/ml ionomycin. MG132 (10 *μ*M) was added during the last 1 h of incubation. (**i**) FACS-sorted (GFP+ and GFP−) spleen cells from A1 shRNA knockdown mice were treated for 8 h with 2 *μ*g/ml Con A. GFP− indicates control cells; GFP+ indicates spleen cells with significant A1 knockdown. In (**b**–**d**) and (**f**–**i**) cell lysates were prepared, western blotted and probed with the A1 monoclonal antibody or antibodies to HSP70 or actin (used as a protein loading control)

## References

[bib1] 1Hatakeyama S et alInt Immunol 10: 631–637.9645611

[bib2] 2Ottina E et alBlood 119: 6032–6042.22581448

[bib3] 3Smits C et alStructure 16: 818–829.18462686

[bib4] 4Herold MJ et alJ Immunol 168: 3902–3909.11937545

[bib5] 5Herold MJ et alJ Biol Chem 281: 13663–13671.

[bib6] 6Grumont RJ et alGenes Dev 13: 400–411.10049356

[bib7] 7Ulleras E et alBlood 111: 3081–3089.18182578

[bib8] 8Lin EY et alJ Immunol 151, 1979-1988.

[bib9] 9Verschelde C et alCell Death Differ 10: 1059–1067. 10.1038/sj.cdd.4401265 12934080

